# Supplementary figures and images for: Larger active site in an ancestral hydroxynitrile lyase increases catalytically promiscuous esterase activity
Source: PLoS One. 2020 Jun 30;15(6):e0235341. doi: 10.1371/journal.pone.0235341 (PMC7326234; doi:10.1371/journal.pone.0235341)

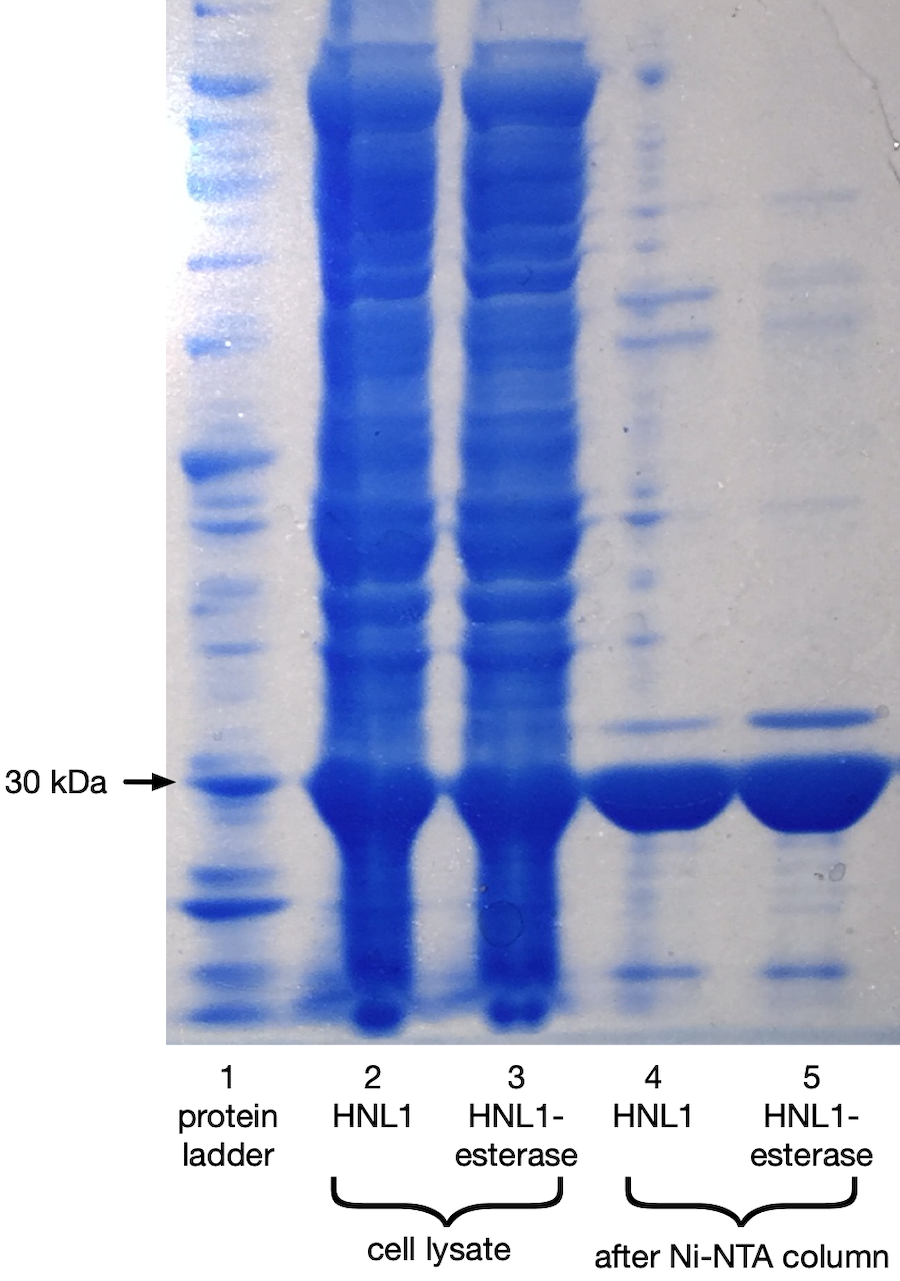

Supplement: S1 Fig — Lane 1 is a protein ladder with an arrow marking the 30 kDa band. Lanes 2 and 3 are the cell lysate for HNL1 and HNL1-esterase catalysis variant, respectively. Lanes 4 and 5 are the purified proteins after Ni-NTA chromatography for HNL1 and HNL1-esterase catalysis variant, respectively. The strong bands at ~30 kDa in lanes 2–5 correspond to the desired proteins (expected molecular weight 31 kDa). (TIF) [file pone.0235341.s001.tif]

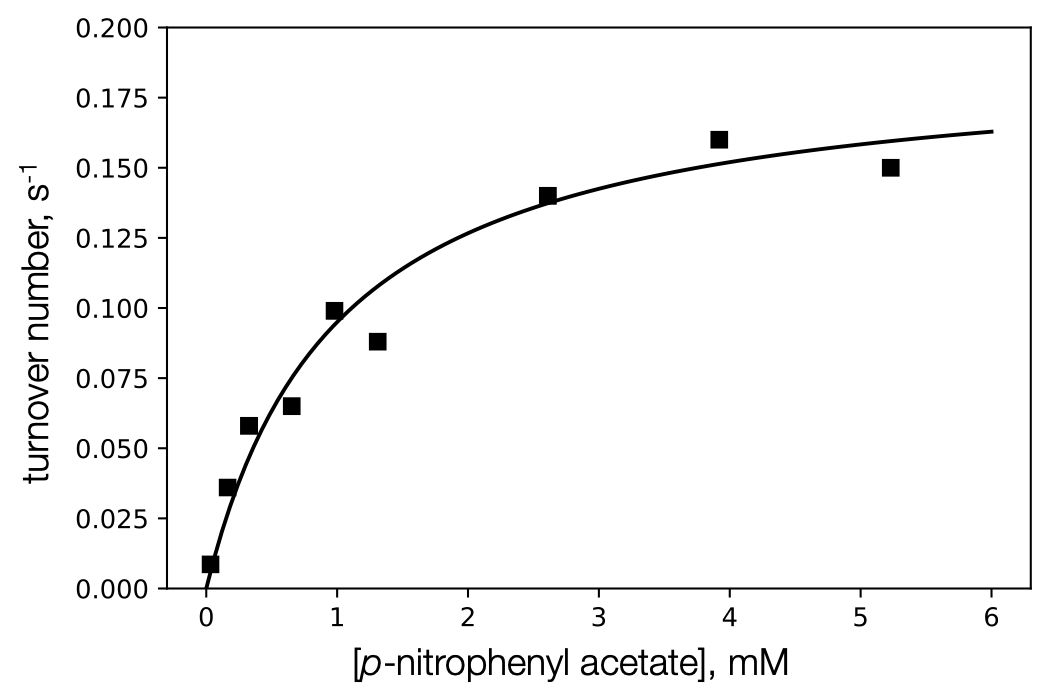

Supplement: S2 Fig — Squares are experimental data and the line is the best fit to the Michaelis-Menten equation where KM = 1.0±0.2 mM and kcat = 0.19±0.01 s-1 or 11±1 min-1. (TIF) [file pone.0235341.s002.tif]

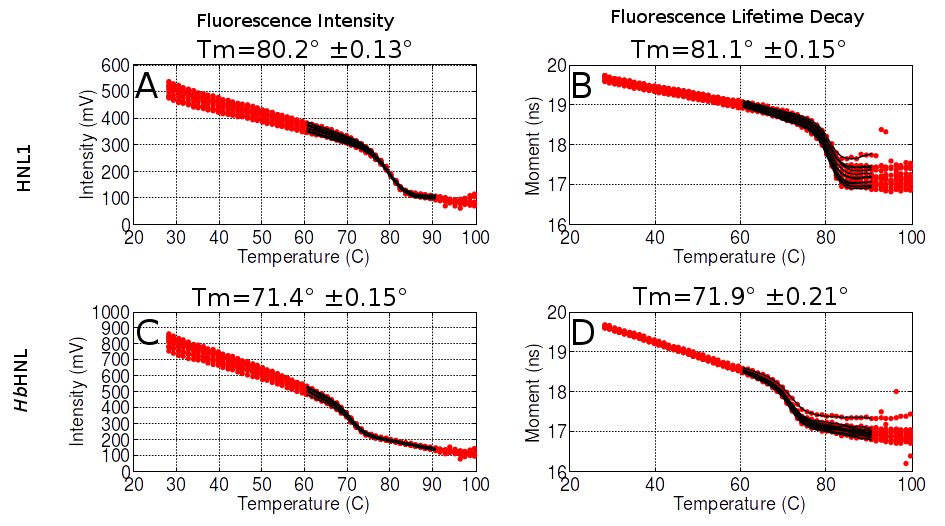

Supplement: S3 Fig — Melting points were measured by decreases in fluorescence intensity (A & C) and by decreases in fluorescence lifetime (B & D). The temperature was increased from ambient temperature to 100 °C at 1 °C /min while monitoring the fluorescent properties. The data for 12 replicates of each protein (red) were fit to a theoretical melting curve (black). The average Tm and standard deviation are shown above each graph. (TIF) [file pone.0235341.s003.tif]

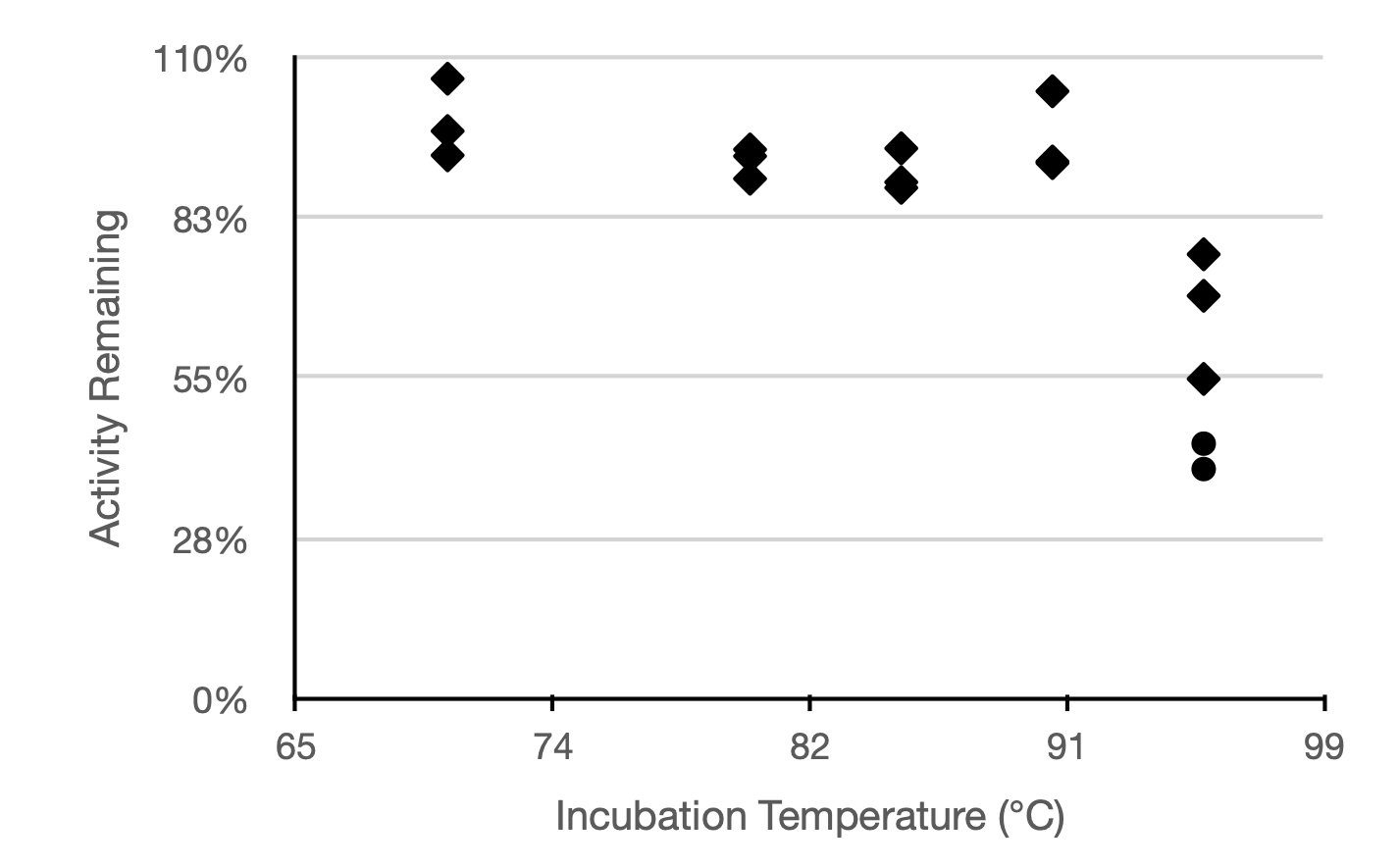

Supplement: S4 Fig — Samples were heated at the indicated temperature for 15 min (diamonds) or 30 min (circles), cooled on ice for 15 min, then assayed for HNL activity at room temperature. The activity is relative to the activity of unheated samples. Reducing the activity of HNL1 by 50% required 30 min at 95 °C. (TIF) [file pone.0235341.s004.tif]

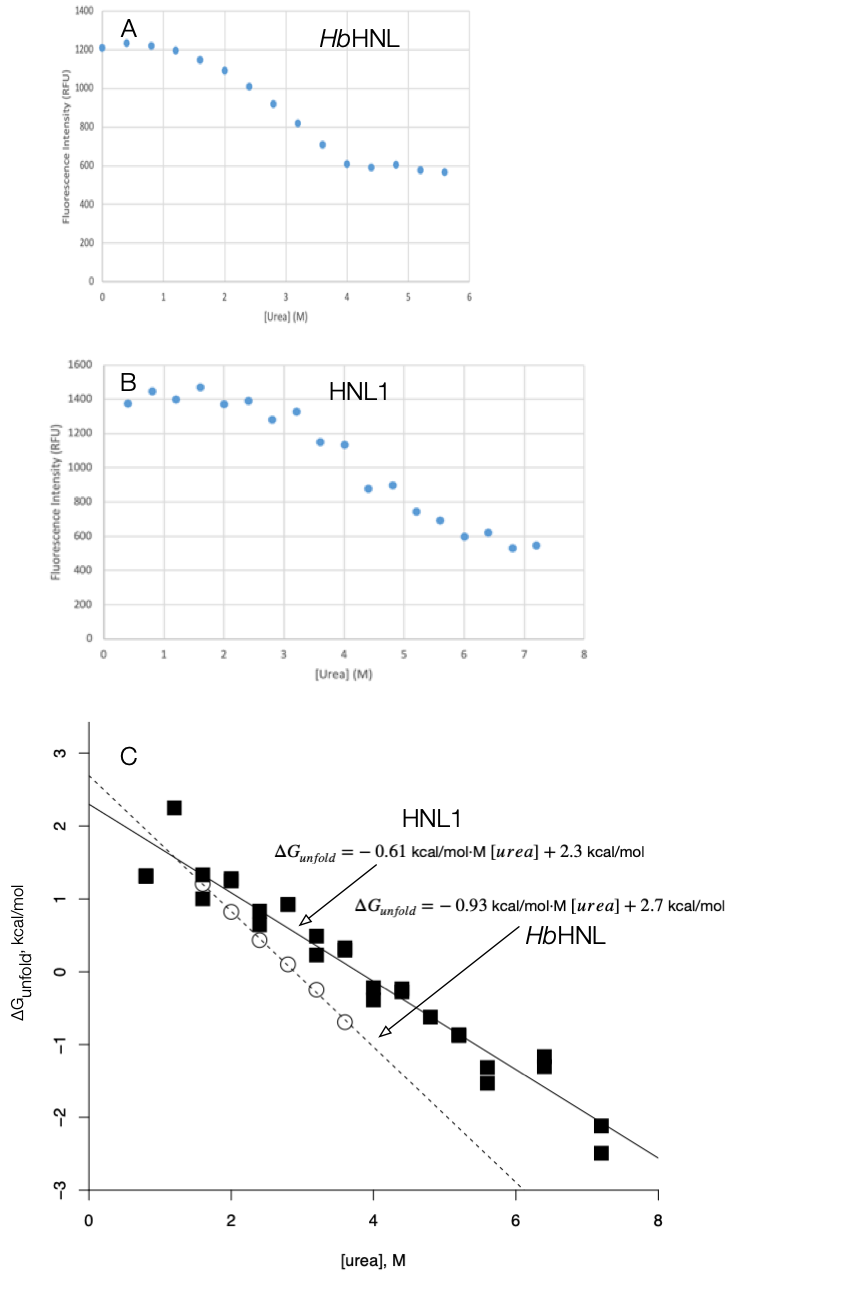

Supplement: S5 Fig — The fluorescence of HbHNL (panel A) and HNL1 (panel B) decreased in solutions of increasing urea concentrations as the proteins unfold. Half of the HbHNL unfolded at 2.8 M urea, while unfolding half of the HNL1 required 3.9 M urea. (C) The slope of the free energy of unfolding versus urea concentration plot is shallower for HNL1 (filled squares, m-value = 0.61 kcal/mol·M) than for HbHNL (open circles, m-value = 0.93 kcal/mol·M) indicating that HNL1 unfolds less completely than HbHNL. The y-intercept corresponds to the free energy of unfolding in pure water. Since the slope differs significantly, the difference in the intercepts is not a good measure of difference in stability. (TIF) [file pone.0235341.s005.tif]

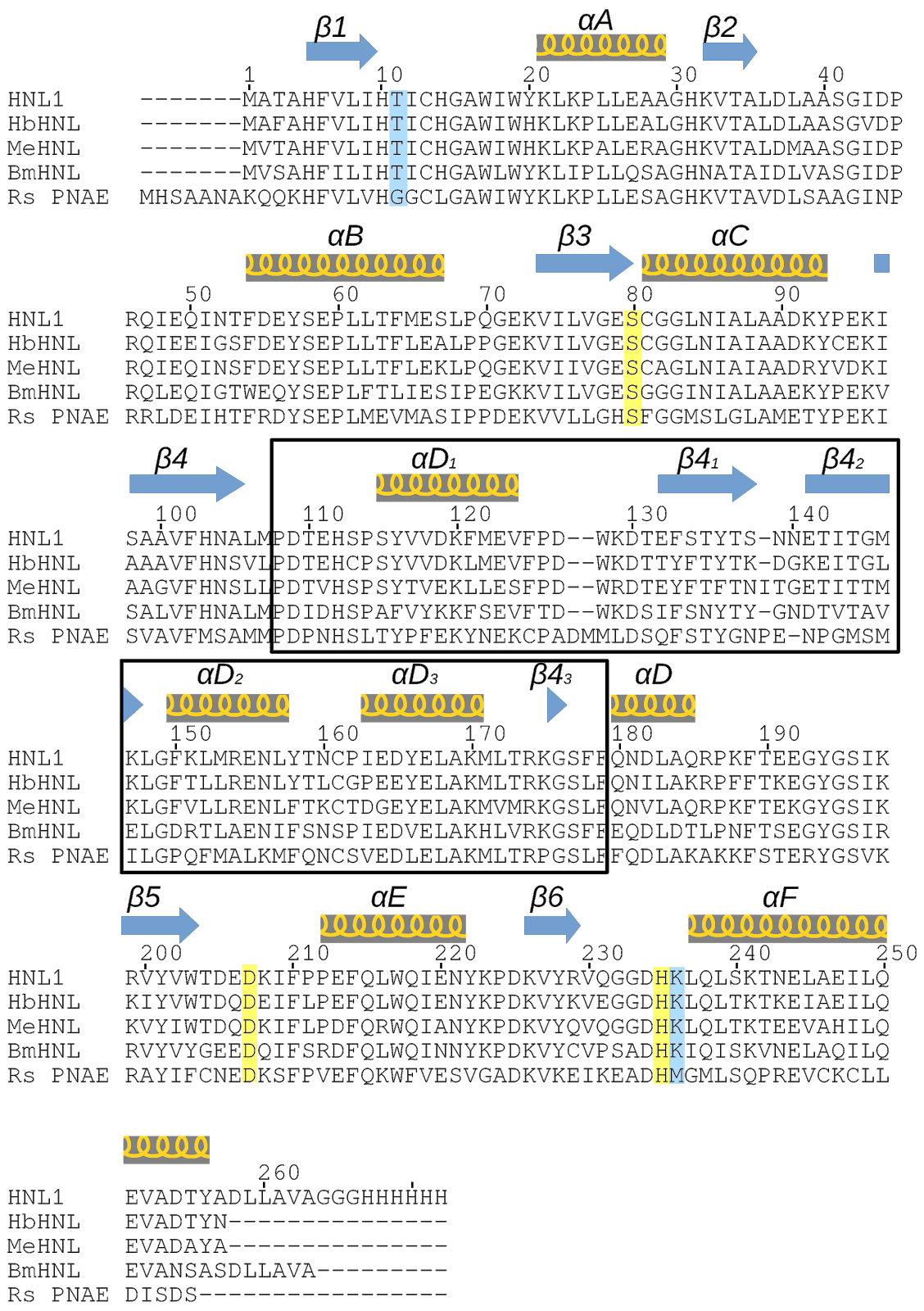

Supplement: S6 Fig — The modern proteins are hydroxynitrile lyases HbHNL (P52704.1 pdb id 1yb6), MeHNL (P52705.3 pdb id 1dwo), BmHNL (BAI50630.1 pdb id 3wwo), and esterase from Rauvolfia serpentina, RsPNAE (Q9SE93.1 pdb id 2wfl). Yellow highlights the conserved catalytic triad of Ser, His, and Asp and blue highlights the threonine and lysine, which also contribute to HNL catalysis. Like other esterases, RsPNAE has a glycine in place of threonine and methionine in place of lysine. The cap domain residues are boxed. Secondary structural elements of HNL1 are indicated above alignment and are also similarly positioned in the aligned sequences. (TIF) [file pone.0235341.s006.tif]

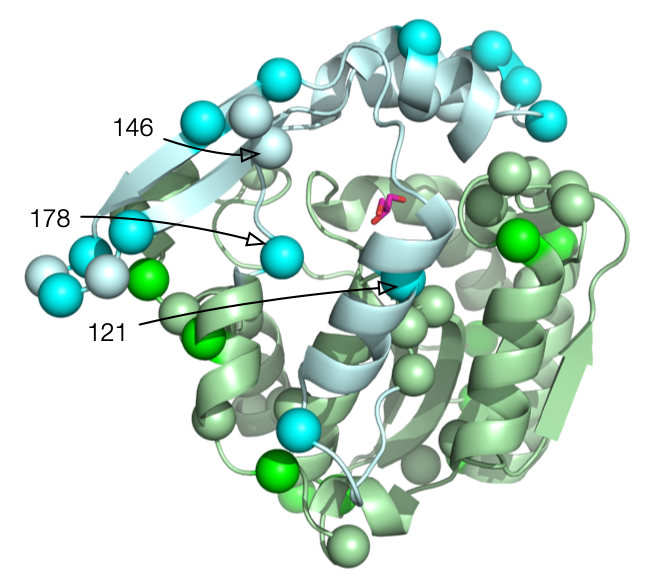

Supplement: S7 Fig — Light green marks the catalytic domain, while light cyan marks the lid domain. The Ca atoms of the differing amino acids are shown as spheres. Conservative substitutions (21 in the catalytic domain, 6 in the lid domain) are in the same color as the corresponding ribbon, while non-conservative substitutions (10 in the catalytic domain, 12 in the lid) are in darker colors. The solvent glycerol bound in the active site is in pink and red sticks. The conservative substitutions in the catalytic domain are: 20, 43, 50, 53, 65, 67, 88, 98, 105, 106, 107, 186, 191, 199, 200, 206, 208, 233, 235, 240, 245; the non-conservative substitutions in the catalytic domain are: 3, 30, 52, 70, 94, 182, 188, 211, 243, 257; the conservative substitutions in the lid domain are: 133, 139, 141, 146, 153, 165; the non-conservative substitutions in the lid domain are: 113, 121, 132, 134, 138, 140, 142, 151, 160, 162, 163, 178. The three substitutions responsible for the larger active site in HNL1 in the lid domain are labelled by residue numbers. The substitution at 146 is a conservative substitution from Leu in HbHNL to Met in HNL1. The substitutions at 121 and 178 are non-conservative substitutions from Leu in HbHNL to Phe in HNL1. (TIF) [file pone.0235341.s007.tif]

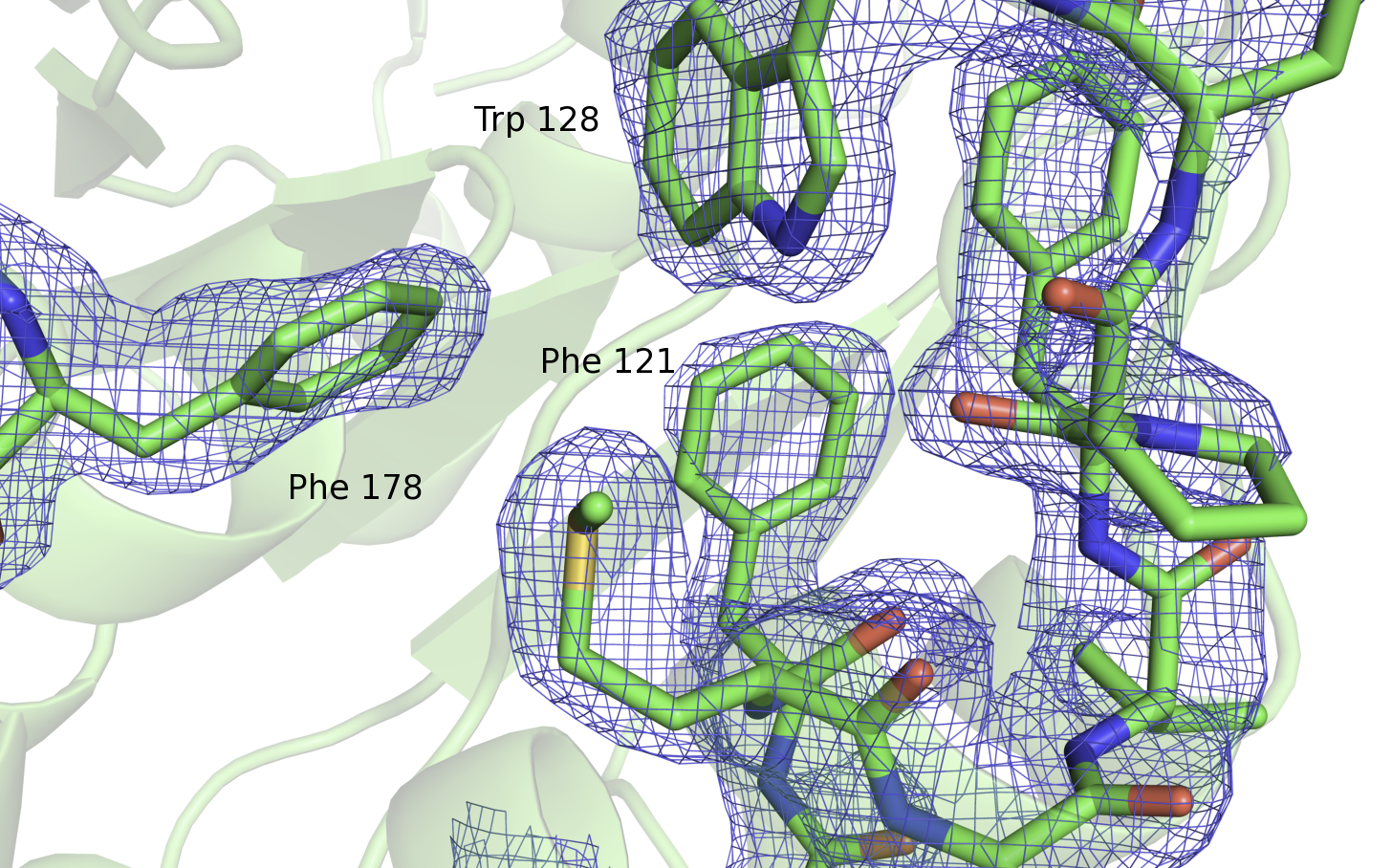

Supplement: S8 Fig — The view is looking into the active site, which is below the plane of the page. Phenylalanines 178 and 121 prevent tryptophan 128 from pushing into the active site. In the modern HbHNL and MeHNL, two leucines replace these two phenylalanines. (TIF) [file pone.0235341.s008.tif]

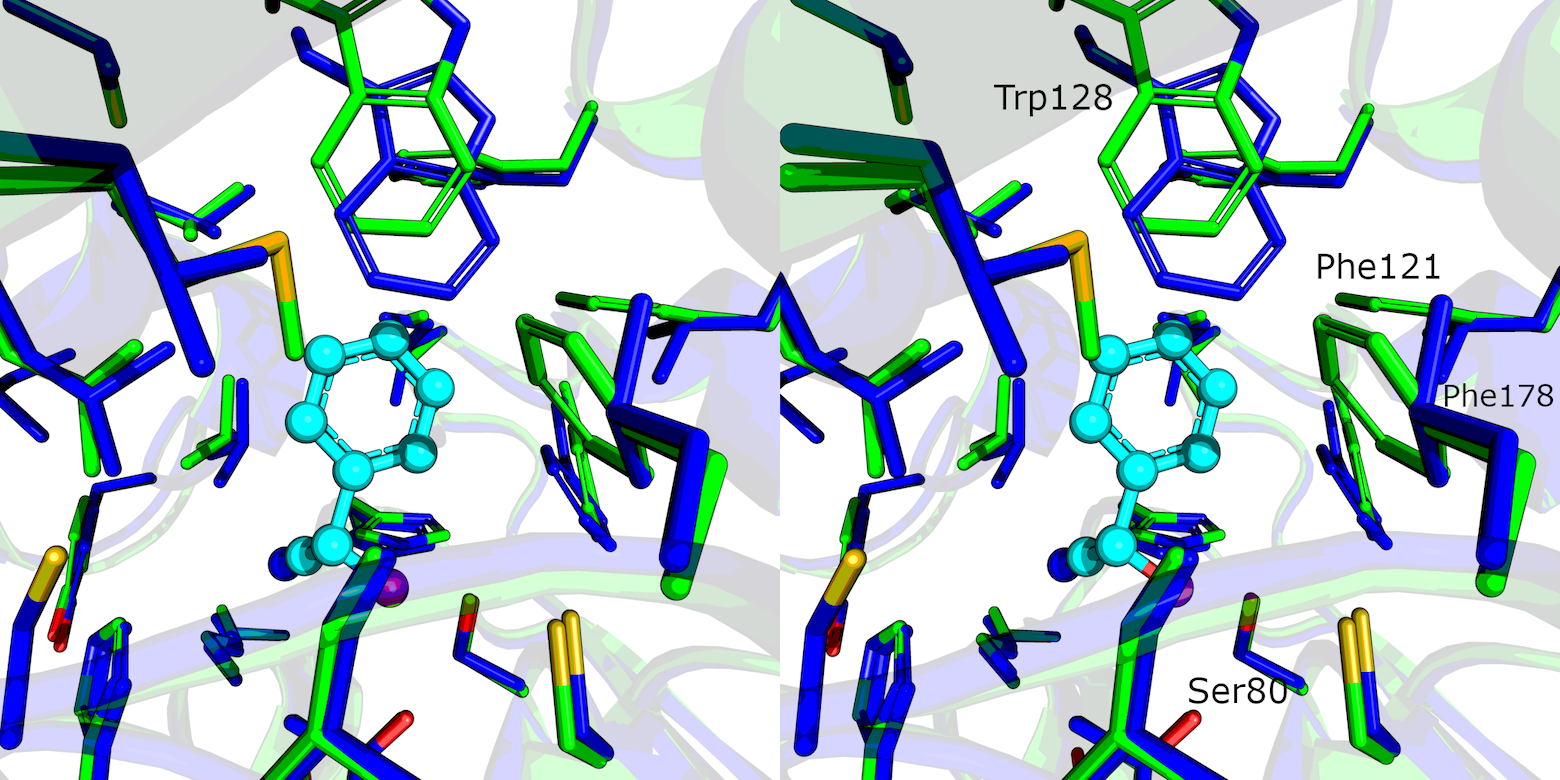

Supplement: S9 Fig — Since the structure of HNL1 (pdb id 5tdx, green) does not contain bound mandelonitrile, the structure of HbHNL (pdb id 3c6x, blue) chosen for comparison also lacks bound mandelonitrile. However, mandelonitrile (cyan balls and sticks) has been added as found in another structure of HbHNL (pdb id 1yb6) to orient the viewer. (TIF) [file pone.0235341.s009.tif]

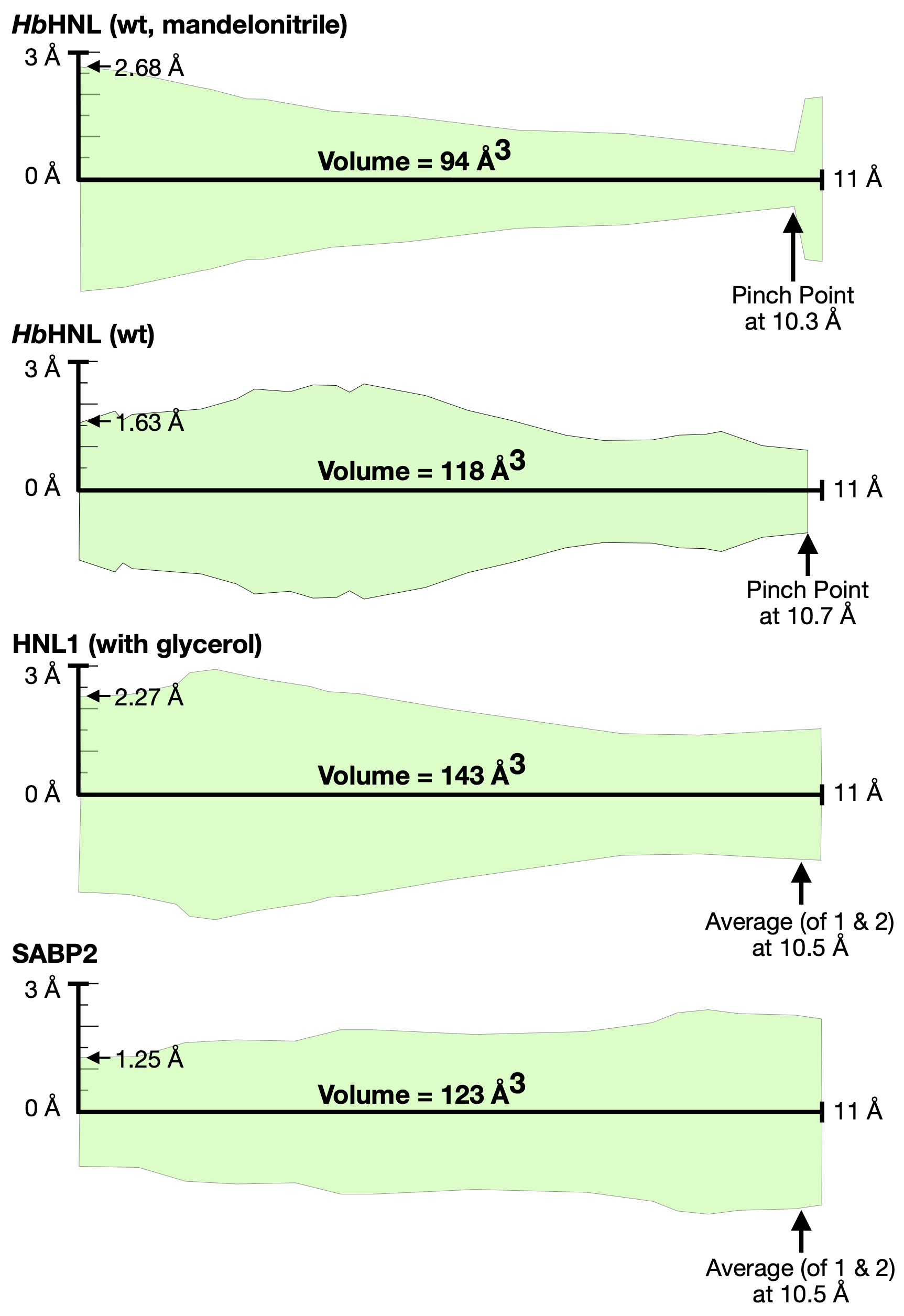

Supplement: S10 Fig — The tunnel leading from the active site to the surface for HbHNL with bound mandelonitrile (pdb id 3c6x) shows a pinch point at 10.3 Å, the tunnel for HbHNL without substrate (pdb id 1yb6) shows a pinch point at 10.7 Å, the tunnel for HNL1 shows a narrowing at ~10.5 Å, and the tunnel for SABP2 with bound product salicylic acid (pdb id 1y7i) shows no narrowing or pinch point. The end of the active site tunnel was defined as 10.5 Å for all enzymes. (TIF) [file pone.0235341.s010.tif]

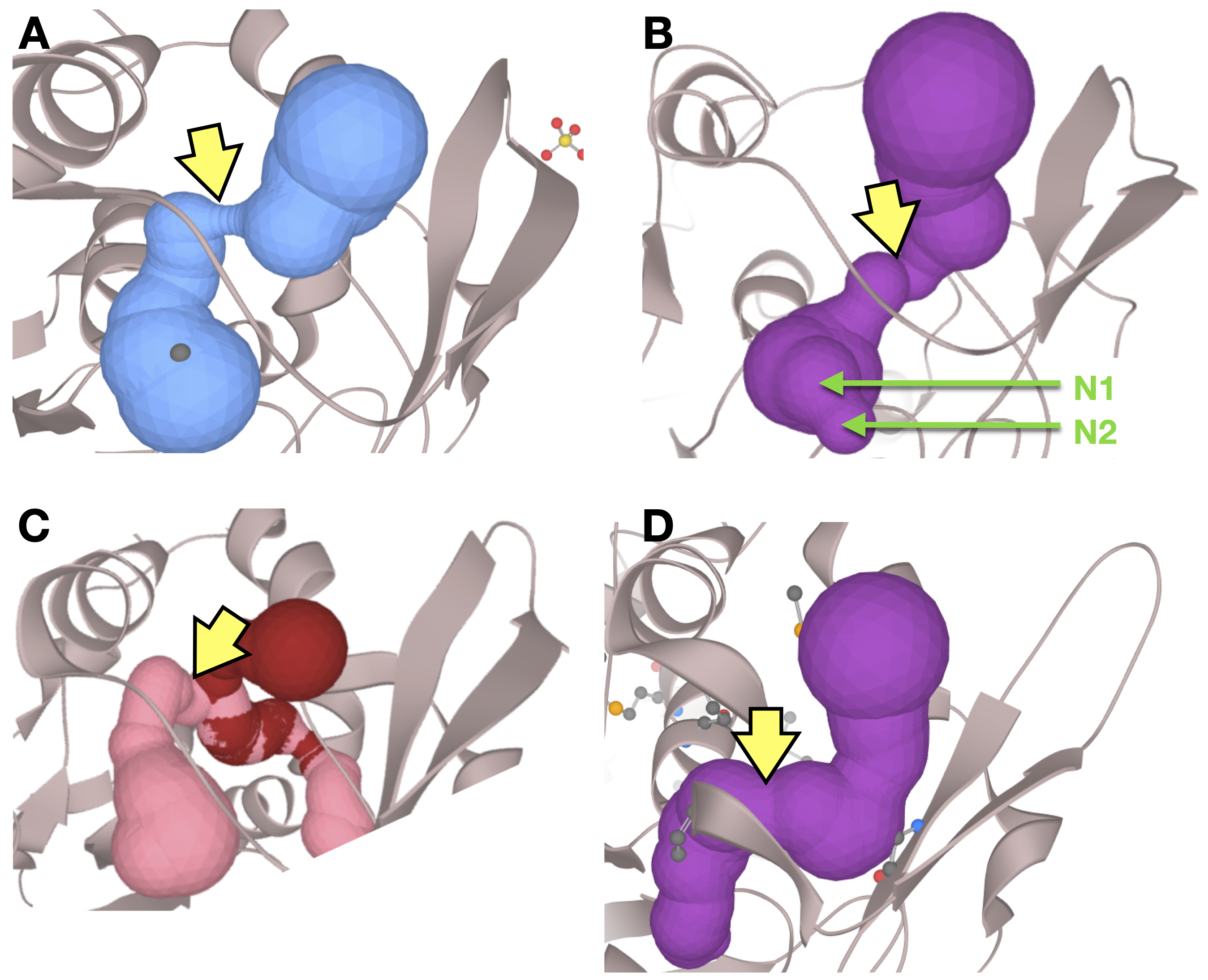

Supplement: S11 Fig — (A) HbHNL with bound mandelonitrile (pdb id 1yb6, MOLEonline tunnel 1), (B) HbHNL without bound substrate (pdb id 3c6x, MOLEonline tunnel 4), (C) HNL1 (pdb id 5tdx, MOLEonline tunnel 11), and (D) SABP2 (pdb id 1y7i, MOLEonline tunnel 4). The same tunnel is compared in each case, although the MOLEonline numbering for each tunnel differed. The start of the active site was defined as the start of the tunnel in HbHNL with bound mandelonitrile. This definition means that for HbHNL without bound substrate, the region labelled N1 in panel B is included, but N2 is not. Yellow arrows indicate the defined end of the active site, based on the consensus pinch points from the bound and unbound HbHNL structures. (TIF) [file pone.0235341.s011.tif]

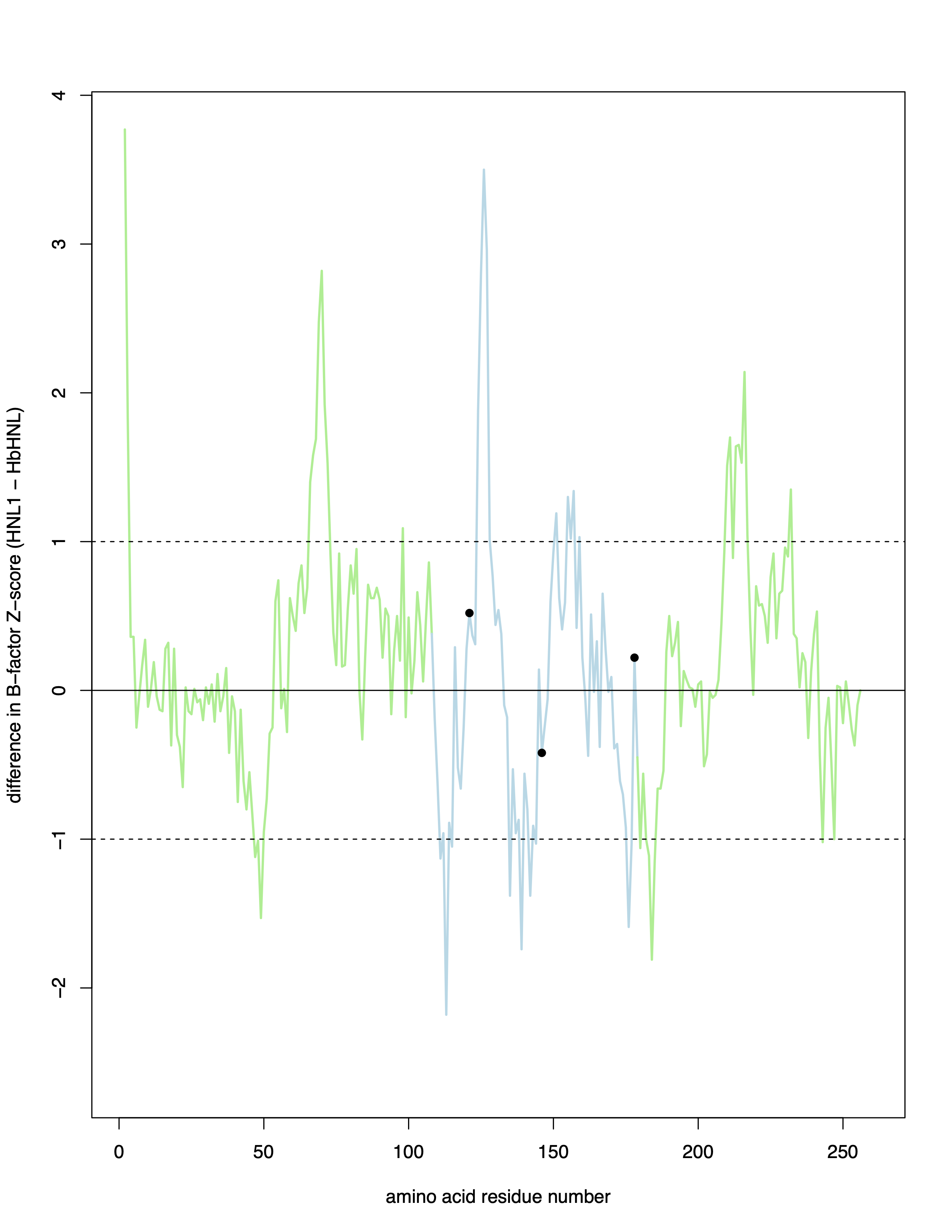

Supplement: S12 Fig — For each structure the B-factors for the C-α atoms were normalized by conversion to Z-scores to identify regions that are more or less mobile than the average. The Z-scores for the HbHNL (pdb id 1sc9) were subtracted from those for HNL1 (pdb id 5tdx, chain A) so positive values indicate regions where HNL1 is more mobile than HbHNL and negative values where HNL1 is less mobile. Differences in Z-score greater than 1 or less than -1 (marked by dotted lines) indicate regions where the difference is larger than one standard deviation of the average B-factor. Regions of the HNL1 structure corresponding to these differences are mapped to the structure in Fig 6. The light green lines indicate the catalytic domain, while the light blue line indicates the lid domain. The three substitutions in HNL1 which expand its active site (Phe121, Met146, Phe178) are indicated as black dots on the plot. The Cα atoms that are more mobile in HNL1 by >1 standard deviation are: 2–3, 66–72, 98, 124–127, 151, 155–157, 159, 210–211, 213–216, 232, 254–256. Those less flexible by >1 standard deviation are: 47, 49, 111, 113, 135, 139, 142, 176, 184–185. (TIF) [file pone.0235341.s012.tif]

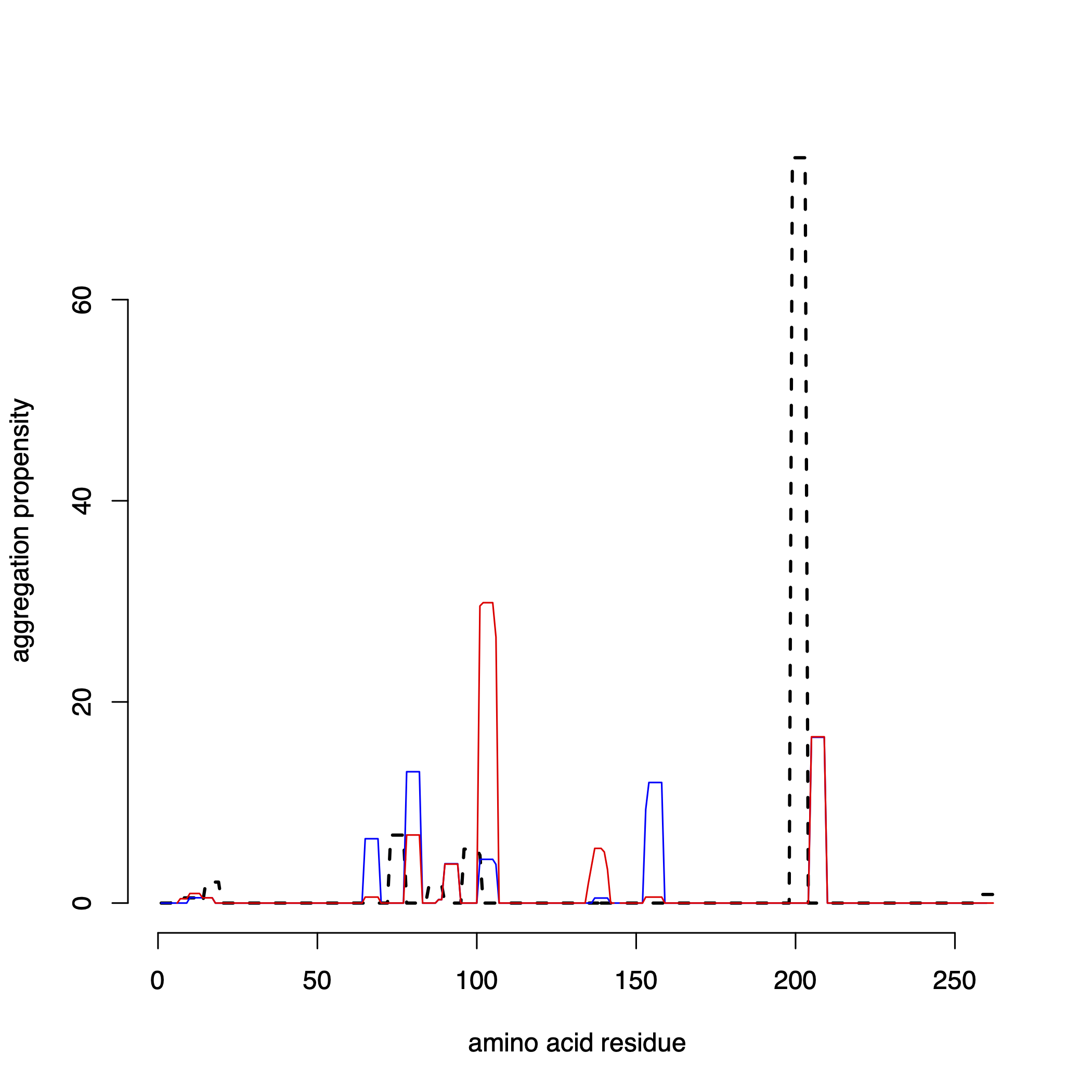

Supplement: S13 Fig — HbHNL (red line), MeHNL (blue line) and HNL1 (dashed black line). All three proteins are predicted to have aggregation-prone regions. Region 200–204 in HNL1 (corresponds to strand β5 in the structure) has the highest predicted aggregation propensity, but experiments showed that HNL1 is less prone to irreversible inactivation than HbHNL and MeHNL. Aggregation propensity was calculated using Tango (http://tango.crg.es) using the conditions 72 °C, pH 7, ionic strength 0.02, 1 mM protein. (TIF) [file pone.0235341.s013.tif]
