# Supplementary material for: Larger active site in an ancestral hydroxynitrile lyase increases catalytically promiscuous esterase activity
Source: PLoS One. 2020 Jun 30;15(6):e0235341. doi: 10.1371/journal.pone.0235341 (PMC7326234; doi:10.1371/journal.pone.0235341)
Supplement: S1 Table — The first three primers pairs are overlapping primers for site-directed mutagenesis using the QuickChange (Agilent) method; the last six primer pairs are non-overlapping, back-to-back primers for site-directed mutagenesis using the Q5 (New England Biolabs) method. “F” designates a forward primer and “R” designates a reverse primer. The mutation site is in bold red. (PDF) [file pone.0235341.s014.pdf]

S1 Table. Mutagenic primers for site directed mutagenesis.

Primer	Mutation	Sequence
HNL1-T11G-F	T11G (ACT→GGC)	5'-CAT TTT GTA TTA ATT CAC **GGC** ATA TGC CAC GGC GCA TGG
HNL1-T11G-R		5'-CCA TGC GCC GTG GCA TAT **GCC** GTG AAT TAA TAC AAA ATG
HNL1-E79H-F	E79H (GAA→CAC)	5'-G GTA ATA CTT GTT GGT **CAC** TCT TGT GGA GGA CTG AAC ATC GCA TTG G
HNL1-E79H-R		5'-C AGT CCT CCA CAA **GAG** TGA CCA ACA AGT ATT ACC TTT TCT CCT TGG G
HNL1-K236G-F	K236G (AAA→GGC)	5'-G GGT GGT GAC CAT **GGC** TTA CAG CTG TCT AAA ACC AAC GAG TTG GCA G
HNL1-K236G-R		5'-GA CAG CTG TAA **GCC** ATG GTC ACC ACC CTG TAC CCT GTA GAC TTT ATC CGG
HNL1-F121L-F	F121L (TTT→TTA)	5'-TA GAT AAA **TTA** ATG GAG GTC TTT CCG
HNL1-F121L-R		5'-CCACATAGGACGGACTG
HNL1-M146L-F	M146L (ATG→TTG)	5'- C ATC ACC GGC **TTG** AAA CTT GG
HNL1-M146L-R		5'- GTCTCGTTATTGCTAGTATACGTGGA
HNL1-F178L-F	F178L (TTT→TTA)	5'- AA GGG AGT **TTA** TTT CAG AAC G
HNL1-F178L-R		5'- TACGCGTCAGCATTTTTG
*Hb*HNL-L121F-F	L121F (CTC→TTC)	5'- C GTG GAT AAG **TTC** ATG GAG GTG
*Hb*HNL-L121F-R		5'- ACGTAAGATGGGCA GTG
*Hb*HNL-L146M-F	L146M (TTA→ATG)	5'- G ATA ACT GGA **ATG** AAA CTG GGC TTC
*Hb*HNL-L146M-R		5'- TCCTTGCCATCTTTAGTGTACG
*Hb*HNL-L178F-F	L178F (TTA→TTT)	5'- AG GGA TCA **TTT** TTT CAA AAT ATT TTA GC
*Hb*HNL-L178F-R		5'- TCCTTGTCAACATCTTCG

The first three primers pairs are overlapping primers for site-directed mutagenesis using the QuickChange (Agilent) method; the last six primer pairs are non-overlapping, back-to-back primers for site-directed mutagenesis using the Q5 (New England Biolabs) method. "F" designates a forward primer and "R" designates a reverse primer. The mutation site is in bold red.
