# Supplementary material for: Larger active site in an ancestral hydroxynitrile lyase increases catalytically promiscuous esterase activity
Source: PLoS One. 2020 Jun 30;15(6):e0235341. doi: 10.1371/journal.pone.0235341 (PMC7326234; doi:10.1371/journal.pone.0235341)
Supplement: S2 Table — (PDF) [file pone.0235341.s015.pdf]

S2 Table. Catalytic activity of ancestral enzyme HNL1 and modern enzymes *Hb*HNL and MeHNL.[a]

Reaction Type	Substrate	HNL1	HbHNL	MeHNL
Cyanohydrin cleavage	acetone cyanohydrin	880 ±70	2,400 ±100	12,600 ±800[b]
	mandelonitrile	340 ±10	1,530 ±130	1,349 ±20
	lactonitrile[b]	5.0 ±0.2	50 ±11	13 ±1
	2-hydroxypentanenitrile[b]	0.5 ±0.1	7.2 ±0.8	0.66 ±0.06
	2-hydroxyhexanenitrile[b]	0.42 ±0.09	24 ±2	1.1 ±0.1
	2-hydroxy-2-(6-methoxynaph-thalen-2-yl) acetonitrile, **1**[c]	1.06 ±0.04	0.64 ±0.05	Not tested
Nitro-aldol cleavage	2-nitro-1-phenyl ethanol	14 ±1	7.2 ±0.3	0.3
Ester hydrolysis	4-nitrophenyl acetate	2.5 ±0.1	0.19 ±0.06	0.060 ±0.005
	methyl salicylate[d]	0.048 ±0.006	0.066 ±0.006	<0.09
	1-naphthyl acetate[e]	0.016 ±0.002	<0.008	<0.4
	2-naphthyl acetate[e]	0.024 ±0.003	<0.008	<0.4

[a] Rates are k_{cat} values in min^{-1} and the errors are standard deviations. Unless otherwise noted all data are taken from Devamani T, Rauwerdink AM, Lunzer M, Jones BJ, Mooney JL, Tan MAO, et al. Catalytic promiscuity of ancestral esterases and hydroxynitrile lyases. J Am Chem Soc. 2016;138: 1046-1056. doi: 10.1021/jacs.5b12209

[b] rate at 5 mM substrate.

[c] This work; rate at 0.45 mM substrate.

[d] rate at 0.5 mM substrate.

[e] rate at 2 mM substrate.
