# Supplementary material for: Larger active site in an ancestral hydroxynitrile lyase increases catalytically promiscuous esterase activity
Source: PLoS One. 2020 Jun 30;15(6):e0235341. doi: 10.1371/journal.pone.0235341 (PMC7326234; doi:10.1371/journal.pone.0235341)
Supplement: S3 Table — (PDF) [file pone.0235341.s016.pdf]

S3 Table. Location and conservative/non-conservative nature of the 49 amino acid differences between HNL1 and *Hb*HNL.[a]

	**entire protein**	**lid domain**	**catalytic domain**
total residues	257 total<br>188 surface, 73.2% of 257<br>69 interior, 26.8% of 257	72 total<br>59 surface, 81.9% of 72<br>13 interior, 18.1% of 72	185 total<br>128 surface, 69.2% of 185<br>57 interior, 30.8% of 185
residues that differ	49, 19.1% of 257<br>40 surface, 81.6% of 49<br>9 interior, 18.4% of 49	18, 25% of 72<br>15 surface, 83.3% of 18<br>3 interior, 16.7% of 18	31, 16.8% of 185<br>25 surface, 80.6% of 31<br>6 interior, 19.4% of 31
conservative replacements	27<br>19 surface, 70.4% of 27<br>8 interior, 29.6% of 27	6, 33% of 18<br>4 surface, 66.7% of 6<br>2 interior, 33.3% of 6	21, 67.7% of 31<br>15 surface, 71.4% of 21<br>6 interior, 28.6% of 21
non-conservative replacements	22<br>21 surface, 95.5% of 22<br>1 interior, 4.5% of 22	12, 66.7% of 18<br>11 surface, 91.7% of 12<br>1 interior, 8.3% of 12	10, 32.3% of 31<br>10 surface, 100% of 10<br>0 interior, 0% of 10

[a]Surface residues are those that have at least 2.5 Å^2 exposed to the solvent in the HNL1 structure calculated using the PyMOL script findSurfaceResidues (http://pymolwiki.org/index.php/FindSurfaceResidues).
