# Supplementary material for: Larger active site in an ancestral hydroxynitrile lyase increases catalytically promiscuous esterase activity
Source: PLoS One. 2020 Jun 30;15(6):e0235341. doi: 10.1371/journal.pone.0235341 (PMC7326234; doi:10.1371/journal.pone.0235341)
Supplement: S4 Table — (PDF) [file pone.0235341.s017.pdf]

S4 Table. Potential structural contributions to thermostability in *Hb*HNL and HNL1.[a]

**property**	***Hb*HNL**	**HNL 1**
Total solvent accessible surface area,[b] $Å^2$	26,722	27,024
Non-polar solvent accessible surface area,[c] $Å^2$	16,894	16,650
Number of electrostatic interactions[d]	111	109
Number of nonpolar contacts[e]	256	262
Number of hydrogen bonds[f]	143	140

[a]Calculations are for one monomer of the protein only without water or bound ligands. *Hb*HNL structure pdb id 1yb6 used in comparison.

[b]Calculated using the get_area function of PyMOL

[c]Non-polar surface area calculated by the exposed surface area of carbon atoms regardless of which residue they occur in.

[d]Number of side chain oxygens in Asp or Glu and side chain nitrogens in Arg, Lys or His with an inter-atomic distance less than 7.0 Å calculated using WHAT IF

[e]Number of interactions between the side chains of Ala, Val, Ile, Leu, Met, Phe, Trp, Pro, or Tyr where the side chain atoms are within 5 Å, Calculated using ProtInter (https://github.com/maxibor/protinter), which is a command line version of PIC: protein interactions calculator.

[f]Calculated optimal hydrogen bond network according to WHAT IF (https://swift.cmbi.umcn.nl/servers/html/index.html).
